# Joint association of physical activity and body weight with subsequent physical and mental functioning: a follow-up study

**DOI:** 10.1186/1471-2458-13-197

**Published:** 2013-03-06

**Authors:** Vivian Lindholm, Jouni Lahti, Ossi Rahkonen, Eero Lahelma, Tea Lallukka

**Affiliations:** 1 Hjelt Institute, Department of Public Health, University of Helsinki, P.O.B. 41, 00014, Helsinki, Finland

**Keywords:** Physical activity, Body mass index, Overweight, Health functioning, Follow-up

## Abstract

**Background:**

Physical inactivity and overweight are major threats to public health. However, it is not well understood to what extent physical activity might counteract the harmful effects of overweight on functioning. Thus, we examined the joint associations of leisure-time physical activity and body mass index (BMI) with subsequent physical and mental functioning over a follow-up of five to seven years.

**Methods:**

The data were derived from the Helsinki Health Study, which is a cohort study among employees of the City of Helsinki, Finland. The baseline postal survey data were collected among 40-60-year-old employees in 2000–02 (n = 8960, response rate 67%), and the follow-up data in 2007 among all baseline survey respondents (n = 7332, response rate 83%). We divided the participants into six groups according to their amount of physical activity (inactive, moderately active and highly active) and their relative weight (normal weight and overweight). Highly active normal-weight participants were used as a reference group in all the analyses. Poor functioning was defined as the lowest quartile of the Short Form 36 (SF-36) health survey’s physical and mental component summaries, with the follow-up cut-off point also applied at baseline. We used logistic regression analysis adjusted for age, gender, baseline functioning, smoking, alcohol use, marital status, socioeconomic position and working conditions.

**Results:**

At baseline 48% of the participants were overweight and 11% were inactive. After adjustments inactivity was associated with poor physical functioning at follow-up both among the normal-weight (OR 1.51, 95% CI 1.09-2.10) and overweight (OR 2.02, 95% CI 1.56-2.63) groups. Being overweight regardless of activity level was associated with poor physical functioning. Poor physical functioning was practically equally common among the highly active overweight group and the inactive normal-weight group. After adjustments, for mental functioning, only inactivity among the overweight was associated with poor mental functioning (OR 1.39, 95% CI 1.08-1.80).

**Conclusions:**

Physical activity is likely to be beneficial for physical and mental functioning among both those with overweight and normal weight. However, maintaining normal weight is also important for good physical functioning. Therefore, efforts should be made to recommend people to engage in physical activity regardless of weight.

## Background

Physical inactivity and overweight are growing threats to public health in the developed countries. Previous studies have shown that physical inactivity increases the risk of coronary heart disease, type 2 diabetes, some cancers and mortality [1-3]. Similarly, overweight is associated with increased risk of these diseases and mortality [2,4]. Studies have found that both physical inactivity [5,6] and overweight [7,8] contribute to poor physical functioning. Physical inactivity may also have adverse effects on mental functioning and contribute to mental health problems, such as depression [9-11], whereas the associations between overweight and mental health are equivocal [7,12].

Studies on the impact of both physical activity and body weight on various determinants of health have typically assessed the effects of physical activity and weight separately [2,3,13-15]. A review on the joint associations of physical activity, fitness and fatness with mortality, morbidity and disease risks [16] found that high BMI even with high physical activity increases the risk factors for diabetes and cardiovascular disease compared with normal weight and low physical activity. Most studies on the joint association of physical activity and weight with functioning have been cross-sectional [17-21]. The controlled trials among these [17,18], found that physical activity among the obese is associated with better functioning. A randomised controlled trial from the United States [22] showed that weight loss combined with regular physical activity may be beneficial for maintaining physical functioning. Previous prospective studies examining the joint association of physical activity and weight with functioning have focused on vigorous physical activity only, whereas the effects of low-intensity and moderate-intensity exercise are unknown [23,24].

A few studies have assessed the association of physical activity and weight with mental functioning [17,18,20]. They have shown that physical activity may be beneficial for mental functioning, whereas the association of overweight with mental functioning is fractional. However, they are all limited because of cross-sectional designs or small sample sizes. We lack prospective large-scale studies of women and men, examining the joint association of body weight and physical activity, with subsequent physical and mental functioning.

The aim of this study was to examine the joint association of body weight and leisure-time physical activity with physical and mental functioning, among middle-aged women and men, over a follow-up of five to seven years. We also took into account baseline functioning and further covariates including smoking, alcohol use, socioeconomic position, employment status, marital status and physical and mental strenuousness of work. We expected that physical and mental functioning among highly active normal-weight and overweight participants would be better than that among less active normal-weight and overweight participants.

## Methods

The data were derived from the Helsinki Health Study of employees of the City of Helsinki, Finland, aged 40, 45, 50, 55 or 60 years at baseline. The baseline postal survey data were collected in 2000–02 (n = 8960, response rate 67%), and the follow-up survey data in 2007 (n = 7332, response rate 83%) [25,26]. Participants with missing information on physical activity (n = 64), height or weight (n = 45) and baseline or follow-up functioning (n = 439) were excluded. Those with a body mass index (BMI) 18.5 kg/m^2^ or less were also excluded (n = 64). The analyses included 6720 participants, of whom 81% were women. The proportion of non-responders, at both baseline and follow-up, was somewhat higher among men, younger age groups, lower occupational classes and those with long sickness absence [25]. Our sensitivity analyses showed that those with poor health outcomes were more likely to drop out during follow-up. Overall the data were representative of the target population, and the non-response or attrition is unlikely to bias the analyses of determinants of health outcomes substantially. The Helsinki Health Study was approved by the ethics committees of the Department of Public Health, University of Helsinki, and health authorities of the City of Helsinki, Finland.

### Physical activity

The participants were asked about the average time per week that they had spent on leisure-time or commuting physical activity over the last 12 months. They were first asked to estimate the intensity of their physical activity according to four intensity grades: walking, brisk walking, jogging, and running, or activities equivalent to these. Then they were asked to estimate the average weekly time spent in each intensity grade. Approximate metabolic equivalent tasks (MET) were calculated on the basis of the volume of physical activity [27]. The average weekly MET hours of leisure-time physical activity were obtained by multiplying the weekly hours by the estimated MET value of each intensity grade, and then adding together the MET hours per week of the four different intensity grades. We grouped the participants into inactive, moderate and high physical activity groups. The inactive group did 7 MET hours per week or fewer, equivalent to 500 kilocalories (kcal) per week or fewer. It has been shown that an amount of physical activity less than the minimum recommendation of 2.5 hours of brisk walking per week (approximately 1000 kcal) [28] may be beneficial [5,29]. The moderate activity group did 7–30 MET hours per week, which is approximately equal to 500–2000 kcal per week or three hours’ jogging per week or less. The high activity group did more than 30 MET hours per week, which is the recommended amount of physical activity for healthy weight maintenance [28].

### Body mass index

Relative body weight was assessed by BMI calculated from self-reported weight (kg) divided by self-reported height in metres squared. Participants with BMI of 18.5-25 kg/m^2^ were categorized as normal-weight, and those with BMI of over 25 kg/m^2^ as overweight.

### Physical and mental health functioning

Physical and mental health functioning were measured by the physical (PCS) and mental (MCS) component summaries of the Short Form 36 (SF-36) health questionnaire [30]. The SF-36 includes eight subscales: physical functioning, role limitations due to physical problems, bodily pain, general health perceptions, mental health, role limitations due to emotional problems, social functioning and vitality. These eight subscales were compressed into the two component summaries. Higher scores indicate better functioning [30]. The subscales are continuous the scores ranging from 0 to 100, with a mean of 50 (standard deviation, SD = 10) in the U S general population. We classified both low physical and mental functioning as the lowest quartile of the component summary scores at follow-up, separately for women and men (PCS 41.9 for women and 46.0 for men, MCS 48.0 for women and 48.6 for men). The follow-up cut-off point for the lowest quartile of functioning was also used baseline. This helps compare the differences between baseline and follow-up functioning. Sensitivity analyses were conducted with separate cut-off points at baseline and follow-up, and the results were similar.

### Baseline covariates

Covariates measured at baseline included age, gender, baseline physical or mental functioning, smoking, alcohol use, marital status, socioeconomic position, and physical and mental strenuousness of work. Employment status was measured at follow-up, with about 70% employed both at the baseline and follow-up. Age was classified into five groups 40, 45, 50, 55 and 60 years at baseline. Smoking was divided into current smokers and non-smokers. Alcohol use was measured by binge drinking, which implied drinking more than six doses on a single occasion once a month or more [31]. Marital status was classified into partnership or no partnership. Socioeconomic position was divided into four occupational social classes: manual workers, routine non-manual employees, semi-professionals and managers, and professionals. Occupational social class was derived from the employer’s personnel register for those who had given informed consent to such linkage (78%), and completed from the questionnaire data. Physical and mental strenuousness of work was classified into very or fairly light, and fairly or very heavy. The covariates are described in more detail elsewhere [26,31].

### Statistical methods

We analysed the joint association of body weight and physical activity by dividing the participants into six groups: (1) high activity (MET >30 h/week) normal-weight (BMI ≤25 kg/m^2^), (2) moderate activity (MET 7–30 h/week) normal-weight, (3) inactive (MET ≤ 7 h/week) normal-weight, (4) high activity overweight (BMI >25 kg/m^2^), (5) moderate activity overweight and (6) inactive overweight. High activity normal-weight participants were used as a reference group in all the analyses. First we examined the joint association of weight and physical activity with physical and mental functioning, both at baseline and follow-up, using cross-tabulation. There were no significant interactions between the genders (P = 0.65 for physical functioning and P = 0.12 for mental functioning), and pooled data were used for the main analyses. We used logistic regression analysis to examine the effects of the covariates on the studied associations. Age and gender were adjusted for in model 1. Model 2 was additionally adjusted for baseline functioning. The effects of the other covariates were first studied one covariate at a time. However, because their contribution to the examined association was mainly small, all covariates were simultaneously adjusted for in model 3. The results are presented as odds ratios (ORs) and 95% confidence intervals (95% CI). We used SPSS version 18.0.

## Results

At baseline, 48% of the study population were overweight, 11% were physically inactive, 51% were moderately active and 38% were highly active (Table 1). Men were more often overweight (59%) and highly physically active (43%) than women (45% overweight, 36% highly physically active). Men were also more often binge drinkers (24%) than women (7%). Seventy-five per cent of the study population were working at follow-up, and 22% were smokers at baseline.

**Table 1:** Baseline participant characteristics

	**Women**	**Men**	**All**
	**% (n)**	**% (n)**	**% (n)**
**Age** (mean)	49.3	50.5	49.5
**Overweight (**BMI > 25 kg/m^2^)	45	59	48
**Highly physically active** (>30 MET-h/week a)	36	43	38
**Moderately physically active** (7–30 MET-h/week)	54	45	51
**Inactive** (≤ 7 MET-h/week)	10	12	11
**Weight/activity groups**			
high activity(>30 MET-h/week) normal-weight(≤25 kg/m^2^)	24	20	23
moderate activity(7–30 MET-h/week) normal-weight	26	18	25
inactive (≤7 MET-h/week) normal-weight	4	3	4
high activity overweight(>25 kg/m^2^)	12	23	15
moderate activity overweight	26	27	27
inactive overweight	6	9	7
**Socioeconomic status**			
Manual worker	14	25	16
Routine non-manual	40	10	33
Semi-professional	20	20	20
Managers/professional	27	45	30
**Marital status** (in a partnership)	68	79	70
**Employment status** (working) b	76	72	75
**Work physical strenuousness**			
very/fairly light	61	85	65
fairly/very heavy	39	15	35
**Work mental strenuousness**			
very/fairly light	24	27	25
fairly/very heavy	76	74	75
**Smoking**	21	25	22
**Alcohol binge** (drinking)	7	24	10
**Total n = 6720**	81 (5455)	19 (1265)	100 (6720)

a MET = approximate metabolic equivalent task.

b At follow-up.

Baseline physical inactivity and overweight were associated with poor physical functioning, both at baseline and follow-up (Table 2). At follow-up, after adjustment for age and gender, 17% (95% CI 14.7-18.9) of the highly active normal-weight group had poor physical functioning, whereas 37% (95% CI 33.5-41.3) of the overweight inactive group had poor physical functioning. Of the highly active overweight group, a larger proportion (26%, 95% CI 23.4-28.8) had poor physical functioning at follow-up, than the moderately active normal-weight group (19%, 95% CI 17.3-21.3). Instead poor physical functioning among the highly active overweight group and the inactive normal-weight group was almost equally common. Physical functioning tended to decline more in the inactive and overweight groups. However, differences between the groups remained largely similar during follow-up.

**Table 2:** Prevalence (%) of poor and mean physical functioning at baseline and follow-up adjusted for age and gender

	**2000-2002**				**2007**				**% Difference**^**1**^
	**Poor physical functioning**	**95% CI**	**Mean functioning**	**95%CI**	**Poor physical functioning**	**95% CI**	**Mean functioning**	**95%CI**	
	**%**	**%**			**%**	**%**			**%**
high activity normal-weight (n = 1551)	11	9.3-13.0	51.6	51.3-52.0	17	14.7-18.9	49.9	49.5-50.4	+5.6
moderate activity normal-weight (n = 1680)	14	12.6-16.2	50.2	49.9-50.6	19	17.3-21.3	48.8	48.4-49.3	+4.9
inactive normal-weight (n = 272)	20	15.3-24.2	49.0	48.1-50.0	27	21.6-31.6	48.2	47.1-49.3	+6.8
high activity overweight (n = 977)	17	14.9-19.7	49.0	48.5-49.5	26	23.4-28.8	47.2	46.6-47.7	+8.8
moderate activity overweight (n = 1795)	26	24.2-27.7	47.2	46.8-47.6	33	30.8-34.7	45.4	45.0-45.9	+6.7
inactive overweight (n = 445)	30	26.0-33.0	46.0	45.3-46.8	37	33.5-41.3	44.3	43.5-45.2	+7.9
total (n = 6720)	18		49.2		25		47.6		+6.4

^1^% difference in poor physical functioning between baseline and follow-up.

In mental functioning, after adjustment for age and gender, the proportion of those with poor functioning was higher among the inactive groups, both at baseline and follow-up (Table 3). Of the inactive groups, normal-weight 29% (95% CI 24.0-34.3) and overweight 32% (95% CI 27.9-35.9) participants, had poor mental functioning at follow-up, whereas the corresponding figures among the highly active groups were 22% (95% CI 19.7-24.0) and 23% (95% CI 20.5-25.9). The change in mental functioning during follow-up was small in all groups.

**Table 3:** Prevalence (%) of poor and mean mental functioning at baseline and follow-up adjusted for age and gender

	**2000-2002**				**2007**				**% Difference**^**1**^
	**Poor mental functioning**	**95% CI**	**Mean functioning**	**95%CI**	**Poor mental functioning**	**95% CI**	**Mean functioning**	**95%CI**	
	**%**	**%**			**%**	**%**			**%**
high activity normal-weight (n = 1551)	21	19.0-23.3	52.6	52.1-53.1	22	19.7-24.0	52.8	52.3-53.3	+0.7
moderate activity normal-weight (n = 1680)	27	25.1-29.3	50.8	50.4-51.3	25	23.2-27.3	51.8	51.3-52.3	−1.9
inactive normal-weight (n = 272)	31	25.7-36.0	50.1	48.9-51.2	29	24.0-34.3	51.2	50.0-52.3	−1.7
high activity overweight (n = 977)	22	19.4-24.8	53.0	52.4-53.6	23	20.5-25.9	52.8	52.1-53.4	+1.1
moderate activity overweight (n = 1795)	27	24.6-28.6	51.8	51.4-52.3	26	23.8-27.8	51.8	51.3-52.2	−0.8
inactive overweight (n = 445)	31	27.3-35.4	50.4	49.5-51.3	32	27.9-35.9	50.7	49.8-51.6	+0.6
total (n = 6720)	25		51.8		25		52.1		−0.4

^1^% difference in poor mental functioning between baseline and follow-up.

In logistic regression analyses, after adjustment for age and gender, being active and overweight was associated with poor physical functioning at follow-up (Table 4, OR 3.05, 95% CI 2.40-3.87). Adjusting for baseline physical functioning attenuated the association (OR 2.11, 95% CI 1.63-2.73). Adjusting simultaneously for all the other covariates (model 3) had only a small impact on the association (OR 2.02, 95% CI 1.56-2.63). After adjustments both being overweight and highly active (Table 4, OR 1.48, 95% CI 1.19-1.84), and being normal-weight and inactive (OR 1.51, 95% CI 1.09-2.10) were equally associated with poor physical functioning.

**Table 4:** Joint association of baseline physical activity and relative weight with poor physical functioning at follow-up

	**Model 1**		**Model 2**		**Model 3**	
	**OR**	**95% CI**	**OR**	**95% CI**	**OR**	**95% CI**
high activity normal-weight (n = 1551)	1		1		1	
moderate activity normal-weight (n = 1680)	1.21	1.01-1.45	1.10	0.91-1.34	1.10	0.90-1.33
inactive normal-weight (n = 272)	1.83	1.35-2.48	1.54	1.11-2.13	1.51	1.09-2.10
high activity overweight (n = 977)	1.78	1.46-2.18	1.53	1.24-1.90	1.48	1.19-1.84
moderate activity overweight (n = 1795)	2.45	2.07-2.91	1.85	1.54-2.22	1.79	1.49-2.16
inactive overweight (n = 445)	3.05	2.40-3.87	2.11	1.63-2.73	2.02	1.56-2.63

Model 1 adjusted for age and gender.

Model 2 1 + baseline physical functioning.

Model 3 2 + marital status, socioeconomic status, alcohol binge drinking, smoking, work physical and mental strenuousness and employment status at follow-up.

OR = odds ratio and 95% CI = 95% confidence intervals from logistic regression analysis.

After adjustment for age and gender, physical inactivity was associated with poor mental functioning among the normal-weight (Table 5, OR 1.46, 95% CI 1.10-1.95) and the overweight (OR 1.68, 95% CI 1.33-2.12). Adjustment for baseline mental functioning attenuated the association (OR 1.29, 95% CI 0.95-1.75 for normal-weight and OR 1.46, 95% CI 1.13-1.88 for overweight participants). After full adjustments the association disappeared among the normal-weight (OR 1.25, 95% CI 0.92-1.70), but remained among the overweight (OR 1.39, 95% CI 1.08-1.80). The mental functioning was poorer among the overweight inactive participants, while the mental functioning among the moderately and highly active overweight participants was approximately the same.

**Table 5:** Joint association of baseline physical activity and relative weight with poor mental functioning at follow-up

	**Model 1**		**Model 2**		**Model 3**	
	**OR**	**95% CI**	**OR**	**95% CI**	**OR**	**95% CI**
high activity normal-weight (n = 1551)	1		1		1	
moderate activity normal-weight (n = 1680)	1.20	1.02-1.42	1.09	0.92-1.30	1.07	0.90-1.28
inactive normal-weight (n = 272)	1.46	1.10-1.95	1.29	0.95-1.75	1.25	0.92-1.70
high activity overweight (n = 977)	1.08	0.89-1.30	1.06	0.86-1.30	1.05	0.85-1.29
moderate activity overweight (n = 1795)	1.24	1.05-1.46	1.13	0.95-1.34	1.09	0.91-1.30
inactive overweight (n = 445)	1.68	1.33-2.12	1.46	1.13-1.88	1.39	1.08-1.80

Model 1 adjusted for age and gender.

Model 2 1 + baseline mental functioning.

Model 3 2 + marital status, socioeconomic status, alcohol binge drinking, smoking, work physical and mental strenuousness and employment status at follow-up.

OR = odds ratio and 95% CI = 95% confidence intervals from logistic regression analysis.

## Discussion

In this study we examined the joint association of physical activity and relative bodyweight with subsequent physical and mental functioning, among middle-aged employees of the City of Helsinki, over a follow-up of 5–7 years. We found that both overweight and physical inactivity jointly contributed to poor physical functioning, although weight tended to dominate the association somewhat. Thus high physical activity at baseline may lead to better physical functioning at follow-up, both among those of normal weight and the overweight, whereas overweight contributes to poor physical functioning even among those who are highly active. The highly active overweight and the inactive normal-weight, were equally associated with poor physical functioning, with those who where inactive and overweight being associated most strongly with poor physical functioning. In mental functioning, physical inactivity tended to dominate the joint association between physical activity and body weight. Adjusting for baseline physical or mental functioning attenuated the association between weight and physical activity with functioning at follow-up, but the associations remained in the overweight inactive group.

There is previous evidence that physical activity and weight maintenance both contribute to better physical functioning [17-24]. This is in accordance with our study, although we found that weight somewhat dominates the joint association with physical functioning. Most studies [17-21,24] show that physical activity is more important than weight maintenance for maintaining good physical functioning. Only two previous studies [22,23] suggest that the effect size of weight is of a similar magnitude to that of physical activity. Most of these studies [17-21] were cross-sectional and as such unable to show the direction of the association. Physical activity may relate to better physical functioning owing to its muscle-strengthening effects [32] and improvement of balance control [33]. Additionally it may prevent various chronic diseases [1], which undermine physical functioning. Maintaining normal weight may also prevent several diseases [4] and mobility disabilities [34] owed to overweight.

The available evidence suggests [23,24] that the physically active overweight have better physical functioning than their normal-weight counterparts, but in our study the physical functioning of highly active overweight and inactive normal-weight participants was similar. This could result from the use of distinct measures of physical functioning or different cut-off points between weight and activity groups. To further analyse any potential factors we performed several sensitivity analyses. We applied different cut-off points and classifications, but the results remained similar. A potential bias relates to muscular men, who may be classified as overweight according to their BMI. However, this is less of a problem among men in late middle age.

The association of physical activity and weight with mental functioning is more equivocal. A previous study on the present data [7] found that overweight was not associated with mental functioning. Some previous studies [9,35] have not considered weight, but they have shown that physical activity can be beneficial for mental functioning. A cross-sectional controlled trial [18] found that physical activity was associated with better mood and functioning. Another cross-sectional study [20] examining the joint association of physical activity and weight with mental functioning, found that physical activity is more important than weight for mental functioning. It also found that overweight inactive people are at greatest risk of poor mental functioning. This is in accordance with our study, which showed only minor differences in mental functioning between the highly active and the moderately active participants, with the overweight inactive participants being most likely to show poor mental functioning. Another previous study [18] suggested that even small amounts of physical activity can improve mental functioning. The study [18] also showed that normal weight is associated with better mood and functioning. We also found adverse effects of overweight on mental functioning among the inactive.

Except for baseline functioning, none of our covariates had a substantial effect on the associations between weight, physical activity and subsequent functioning. In addition to health behaviours and socio-demographics, we controlled for limiting longstanding illnesses and common mental disorders. We also conducted sensitivity analyses adjusting for the overall quantity of alcohol used. These covariates had negligible effects on the results (data not shown).

Several further sensitivity analyses were conducted. We used different cut-off points for the physical activity and weight groups. For BMI, 27 kg/m^2^ and 30 kg/m^2^ were used as cut-off points. For physical activity, 4 and 14 MET-hours per week were used. We also used different measures for physical functioning, such as the physical functioning (PF) subscale of SF-36, mean values for physical and mental functioning component summaries, and both the lowest quintile and highest quartile of functioning. These analyses did not substantially affect the results reported. Gender stratified analyses were also conducted. However, pooled data were used for the main analyses. Additionally we analysed the effects of vigorous exercise on functioning, as suggested by previous research [13,36]. This was done by analysing both the effects of the amount of physical activity and the intensity of the activity, assessed by practising vigorous physical activity or not. There were practically no differences in the effects of a large amount of physical activity compared with practising vigorous activity or not.

The strengths of this study include the prospective design, the large sample of middle-aged women and men originally employed, and the high participation rate at follow-up. Other strengths are the joint assessment of leisure-time physical activity and weight with functioning, the consideration of several covariates and the several sensitivity analyses. Additionally, identical measures for physical and mental functioning, both at baseline and follow-up, were available. The limitations include self-reported measures. These might cause overestimation of physical activity, overestimation of height, and underestimation of weight [37]. However, it has been shown [24] that self-reported and measured weight and height predict health outcomes broadly in a similar way. BMI is not fully accurate as a measure of overweight, because it cannot distinguish between fat and lean mass. However, the accuracy of BMI has been found sufficient for epidemiological studies [38]. SF-36 is established as a reliable measure of physical and mental functioning [39].

## Conclusions

Physical activity is likely to be beneficial for physical and mental functioning both among those overweight and normal weight. However, maintaining normal weight is also important for maintaining good physical functioning. Within the ageing population, maintaining good physical and mental functioning is one way of preventing disability and subsequent sickness absence [40], as well as disability retirement [41], and thereby helps lengthening work careers. Health and welfare policies should aim at preventing inactivity and overweight, as they have adverse effects on functioning. Efforts should be made to recommend people to engage in physical activity regardless of their body weight.

## Abbreviations

BMI: Body mass index;CI: Confidence interval;Kcal: Kilocalories;MCS: Mental component summary;MET: Metabolic equivalent task;OR: Odds ratio;PCS: Physical component summary;PF: Physical functioning;SD: Standard deviation;SF-36: Short form 36 health survey;SPSS: Statistical package for the social sciences.

## Competing interests

The authors declare that they have no competing interests.

## Authors’ contributions

VL performed the statistical analyses, interpreted the results and drafted the manuscript. JL, OR, EL and TL contributed to designing the study, interpreting results and drafting the manuscript. All authors critically reviewed the manuscript and approved the final version.

## Pre-publication history

The pre-publication history for this paper can be accessed here:

http://www.biomedcentral.com/1471-2458/13/197/prepub
